# Physician Perceptions of the Use of Social Media for Recruitment of Patients in Cancer Clinical Trials

**DOI:** 10.1001/jamanetworkopen.2019.11528

**Published:** 2019-09-18

**Authors:** Mina S. Sedrak, Virginia Sun, Jennifer Liu, Kevin George, Andrew R. Wong, William Dale, Don S. Dizon

**Affiliations:** 1 Department of Medical Oncology and Therapeutics Research, City of Hope, Duarte, California; 2 Department of Population Science, City of Hope, Duarte, California; 3 Department of Supportive Care Medicine, City of Hope, Duarte, California; 4 Lifespan Cancer Institute, Department of Hematology/Oncology, Brown University, Providence, Rhode Island

## Abstract

**Importance:**

Social media campaigns have been successfully implemented in nontherapeutic trials. However, evidence to support their utility in cancer therapeutic trials is limited.

**Objective:**

To examine physician attitudes toward and perceptions of social media use for therapeutic trial recruitment of patients with cancer.

**Design, Setting, and Participants:**

This qualitative study engaged 44 physicians (24 academic based and 20 community based) at the main academic and 6 affiliated community sites of City of Hope in Duarte, California. Semistructured interviews were conducted in person or by telephone from March to June 2018. An interview guide was developed to explore perceptions of social media use for accrual of cancer therapeutic trials. Responses were recorded digitally and transcribed. Data were analyzed using qualitative content analysis.

**Main Outcomes and Measures:**

Physicians’ perceptions of the advantages and disadvantages of using social media for clinical trial recruitment, strategies to improve uptake of social media in clinical trials, and the barriers and facilitators to social media use for professional purposes in general.

**Results:**

Of the 44 participants, 16 (36%) were women, 30 (68%) had more than 10 years of practice experience, 24 (55%) practiced in academia, and 20 (45%) practiced in the community. Physicians most commonly cited increased trial awareness and visibility as an advantage of using social media for trial recruitment. Cited disadvantages were increased administrative burden and risk of misinformation. Physicians also reported a need for institutional-level interventions (eg, restructuring of clinical trial offices to include personnel with social media expertise), increased evidence-based approaches to social media use, and more physician training on the use of social media. Perceived facilitators to professional social media use were networking and education; barriers included lack of time and lack of evidence of benefit.

**Conclusions and Relevance:**

In this qualitative study, physicians recognized the benefits of using social media for clinical trial recruitment but noted that barriers, including increased administrative burden, increased time, and the risk of misinformation, remain. Future interventions to address these concerns are a required first step in increasing digital engagement for clinical trial accrual purposes.

## Key Points

**Question:**

What are physicians’ attitudes toward and perceptions of using social media to recruit participants for cancer clinical trials?

**Findings:**

In this qualitative study of 44 physicians from academic and community practices, physicians cited increased trial awareness and visibility as advantages of social media. Commonly cited disadvantages were increased administrative burden and risk of misinformation.

**Meaning:**

This study’s findings suggest that physicians are aware of the benefits of social media as it relates to clinical trial recruitment, but key barriers remain; tailored interventions to address these concerns would be a required first step in increasing digital engagement among physicians.

## Teaser

This qualitative study examines physician attitudes toward and perceptions of social media use for therapeutic trial recruitment of patients with cancer.

## Introduction

Cancer clinical trials are essential for translating scientific discoveries into new treatments.^1^ However, only 3% to 4% of adults participate in cancer therapeutic trials.^2^ Strategies that inform and educate the public on clinical trials have been linked to improvements in patient recruitment.^3^ For example, web-based interactive videos have been shown to improve patient knowledge and reduce attitudinal barriers.^4^ Efforts to modernize clinical trial recruitment in oncology are increasing.^1,5,6,7^

Recent studies suggest that the effective use of social media platforms might offer a new way to communicate with the public about cancer clinical trials and increase awareness and recruitment.^8,9^ Social media platforms that allow users to create and share content^10,11^ may provide a novel, low-cost, and widely accessible method to disseminate information about cancer clinical trials and facilitate recruitment.^1,8,9,12,13,14^ Studies show that patients and clinicians communicate about cancer and cancer clinical trials on social networking sites.^8,15,16,17,18,19^ Additionally, some studies have demonstrated that social media can be leveraged to increase participation in nontherapeutic trials.^20,21,22^ Researchers successfully used a social media campaign to recruit metastatic breast cancer patients to a translational registry study (the Metastatic Breast Cancer Project),^9,22^ and 2 ongoing tumor registry projects (ie, the Metastatic Prostate Cancer Project and the Angiosarcoma Project)^20,21^ are using social media to recruit and engage with patients.

While social media has been successfully implemented in nontherapeutic trials, the value and direct application of social media in cancer therapeutic trials remains unclear, and the evidence to support its utility as a recruitment strategy is limited.^9,13,23^ In particular, little is known about the perception of social media use in cancer research among physicians, who design and conduct clinical trials and recruit patients to them. Given the central role physicians play in recruitment,^24^ we examined their perspectives on the potential advantages of, disadvantages of, and future interventions for leveraging social media for cancer clinical trial recruitment. In addition, we explored their perceptions of the barriers and facilitators to social media use for professional purposes in general.

## Methods

### Study Design and Setting

Semistructured interviews were conducted with 44 physicians at City of Hope (COH) in Duarte, California, from March to June 2018. City of Hope is a National Cancer Institute–designated comprehensive cancer center, supported by the National Cancer Institute to conduct cancer research, care for patients, and provide cancer information to the medical community and general public. This study was conducted at the Duarte campus (main tertiary care center) and 6 affiliated community sites (ie, Antelope Valley, Colton, Mission Hills, Rancho Cucamonga, South Pasadena, and West Covina) located throughout Southern California. The COH institutional review board deemed this study exempt because interview procedures did not pose any significant risk to participants that would require institutional review board oversight. Findings were reported using the Consolidated Criteria for Reporting Qualitative Research (COREQ) reporting guideline.^25^

### Participant Selection

Participants were recruited from academic and community sites to capture a range of physicians’ perspectives across diverse practice settings. Physicians were invited via email to participate in telephone or face-to-face interviews. We initially approached 24 academic physicians and 23 community physicians. Among the 47 approached, 44 physicians (24 from academia and 20 from community sites) agreed to participate and completed the interview; 1 person declined owing to lack of time to complete the interview, and 2 people did not respond to the email invitation.

### Data Collection

Semistructured interviews were conducted with all participants individually. We conducted 24 face-to-face interviews in private rooms in COH medical offices and 20 interviews over the telephone at the participants’ request. All interviews were conducted by 1 of us (K.G.), a trained clinical research coordinator and male postgraduate student with 2 years clinical research experience in oncology and structured training in qualitative interviewing. Participants were informed of the basis of the research and received a written statement of research, which included all the elements of informed consent, prior to commencement. Participants provided informed consent verbally prior to digital recording of the interviews. Participants were unknown to the interviewer and interviewed once, and flexible use of the interview guide was used regarding the depth of exploration of some issues. No other persons were present during the interview. Field notes were made during and/or after the interviews. All interviews were audio recorded with a digital recorder and transcribed verbatim. Transcription was performed by 1 of us (K.G.) and reviewed for quality control by 2 of us (M.S.S. and A.R.W.). No transcripts were returned to participants for feedback.

The interview guide was developed based on an a priori framework after a literature review that examined social media use by physicians, focusing on oncologists and clinical trials where evident. Questions explored perceptions of social media use for clinical trial recruitment and professional purposes in general. A pilot interview with a physician was recorded and transcribed before the study began to ensure appropriate format and structure. This was not included in the final analysis. The interview guide included prompts (eAppendix in the Supplement).

### Data Analysis

Data was managed using NVivo version 12 qualitative data analysis software (QSR International). Two of us (K.G. and A.R.W.) independently coded interview data using thematic content analysis.^26,27^ Specifically, an inductive approach was taken, where themes were derived from the data. This approach does not involve a predetermined theory or framework. The themes were reviewed by 2 of us independently (M.S.S and V.S.); discordant coding was discussed and adjudicated by consensus. The number of physicians who reported each theme was recorded. Participants were not invited to provide feedback on findings.

To ensure methodological rigor, we conducted the study in the following manner: recorded and transcribed interviews^28^; had regular discussion of findings among the study team^29^; used the interview guide flexibly to allow participants’ perspectives to be explored in depth^28^; collected and managed the data in a clear and transparent manner and by choosing an analysis method appropriate for the data^30^; reported results using direct quotes from interviews^31^; provided detail on the sample and setting^32^; and related findings to other literature.^33^

## Results

Of the 44 participants, 16 (36%) were women, 30 (68%) had more than 10 years of practice experience, 24 (55%) practiced in academia, and 20 (45%) practiced in the community (Table 1). The interviews ranged from 3 to 17 minutes (mean [SD], 8 [3] minutes). Overall, 29 physicians (66%) reported that less than 5% of their patients enrolled in clinical trials (Table 1). Physicians identified advantages and disadvantages of using social media for clinical trial recruitment, strategies to encourage use of social media in clinical trials, and barriers and facilitators to social media use for professional purposes in general (Table 2).

**Table 1.** Characteristics of 44 Participating Physicians

Characteristic	No. (%)		
	Academic (n = 24)	Community (n = 20)	Total (N = 44)
Women	12 (50)	4 (20)	16 (36)
Years in practice			
<5	2 (8)	4 (20)	6 (14)
5-10	5 (21)	3 (15)	8 (18)
>10	17 (71)	13 (65)	30 (68)
Patients in practice, No.			
<500	17 (71)	8 (40)	25 (57)
500-1000	6 (25)	8 (40)	14 (32)
>1000	1 (4)	4 (20)	5 (11)
Patients enrolled in clinical trials, %			
<5	13 (54)	16 (80)	29 (66)
5-10	9 (38)	3 (15)	12 (27)
>10	2 (8)	1 (5)	3 (7)

**Table 2.** Identified Themes and Representative Quotes From Interviews With 44 Physicians Regarding Social Media in Oncology Clinical Trials

Domain	Themes	Representative Quotes
**Advantages, Disadvantages, and Strategies for Using Social Media in Clinical Trial Recruitment**		
Advantages	Visibility and awareness	“Many people are online…. It’s a good way to get access to multiple people out there and… get the message out there.”
	Improved communication	“It could be a very promising vehicle, if patients start to know that [social media] can [be a source of] reliable information… about cancer research.”
	Patient engagement	“The more engaged [patients] are, the more empowered they feel…. [Social media] improves decision making…, patient care…, [and] satisfaction.”
Disadvantages	Increased administrative burden	“I don’t have time for it. I have other things that are more important.”
	Risk of misinformation	“A little bit of misinformation can go a long way…. Controlling the quality of conversation and information… [is] challenging.”
	Lack of guidance	“We need legislation, good guidance, a good practice guideline to make sure we don’t make a mistake.”
	Limited outreach	“You’re missing a lot of people who aren’t on social media.”
Strategies	Institutional support	“I would be very interested if it was through a site not linked directly to me—if it’s through our cancer [center]…, if somebody else is doing it.”
	Evidence	“I do want to see evidence of [social media’s] value [in trial recruitment].”
	Training	“A teaching module: learn this, learn how to use this, do it this way…. I would be open to education.”
**Facilitators and Barriers for Professional Social Media Use**		
Facilitators	Networking	“[Social media can] be a very helpful vehicle for talking with other people in their area and disseminating… research findings”
	Education	“I found [social media] to be a very good way to get information… about new thoughts or ideas or discussion from colleagues.”
	Promotion	“To highlight my own research… to bolster interest and enthusiasm.”
Barriers	Familiarity, time	“I’m not as adept at it… so it would take… training... and time.”
	Uncertainty	“From a professional standpoint, I haven’t seen evidence of benefit.”
	Preference	“I would rather have [patients] hear [my advice] from me directly [in person or via telephone] than hearing it on social media.”
	Blurred boundaries	“I don’t want patients directly contacting me through social media.… There needs to be some fine line, and that gets crossed.”

### Advantages of Using Social Media for Clinical Trial Recruitment

There were 3 main themes identified by physicians as potential advantages of social media use for clinical trial recruitment, as follows: (1) increased visibility and awareness, (2) improved communication, and (3) patient engagement. Most physicians cited social media as a useful platform to increase awareness and visibility of clinical trials, recognizing it as a means to reach large populations and easily spread information about available trials. They described social media as a way to easily disseminate clinical trial information online to patients, caregivers, and other physicians because of the large number of users on these platforms: “Many people are online…. It’s a good way to get access to multiple people out there and… get the message out there” (participant 36). Physicians also perceived social media as a valuable mode of communication, describing social media as a promising opportunity to use technology to improve patient understanding and engagement in clinical research. One physician noted that social media could be a useful resource for patients and caregivers to obtain information about clinical trials, saying that “it could be a very promising vehicle, if patients start to know that [social media] can [be a source of] reliable information… about cancer research” (participant 23). The third most commonly cited benefit was the potential of social media to improve patient experience during the clinical trial process. Physicians believed social media could help increase patient education and engagement: “the more engaged [patients] are, the more empowered they feel…. [Social media] improves decision making…, patient care…, [and] satisfaction” (participant 9).

### Disadvantages of Using Social Media for Clinical Trial Recruitment

Physicians also identified disadvantages of the use of social media for clinical trial recruitment, which centered on 4 main themes, as follows: (1) increased administrative burden, (2) risk of misinformation, (3) lack of guidance, and (4) limited outreach. First, physicians reported feelings of discomfort owing to inexperience with social media platforms or a lack of time for the management of social media tools. One physician stated, “I don’t have time for it. I have other things that are more important” (participant 13). Second, physicians expressed apprehension about the potential of social media to oversimplify trials or spread misinformation. They expressed concerns that information might be misconstrued because of the nature of the internet: *“*a little bit of misinformation can go a long way…. Controlling the quality of conversation and information… [is] challenging” (participant 9). A third theme highlighted the regulatory, ethical, and security and privacy issues regarding social media use. Physicians who recognized the challenge of regulating information on the internet expressed the “need [for] legislation, good guidance, a good practice guideline to make sure we don’t make a mistake” (participant 12). Fourth, physicians expressed hesitation to use social media for trial recruitment because of its limited outreach, fearing that using social media would exclude populations that do not have access to such platforms from participating in clinical trials: “you’re missing a lot of people who aren’t on social media” (participant 2). Some physicians also expressed concerns that the underrepresentation of minority groups and/or older adults with cancer would be exacerbated owing to the limited reach of social media.

### Strategies for Effectively Using Social Media for Clinical Trial Recruitment

When physicians were asked for potential strategies to effectively use social media for clinical trial recruitment, 3 common themes were identified, as follows: (1) institutional support, (2) evidence, and (3) training. The most commonly cited strategy for promoting the use of social media for clinical trial recruitment was the provision of institutional structure and personnel support. Physicians expressed interest in using social media for recruitment if they were provided institutional resources to manage recruitment efforts on social media: “I would be very interested if it was through a site not linked directly to me—if it’s through our cancer [center]…, if somebody else is doing it” (participant 1). The second most commonly cited strategy was the establishment of a methodology to guide physicians on how to use social media effectively, with evidence of its benefit: “I do want to see evidence of [social media’s] value [in trial recruitment]” (participant 23). The third most prominent theme was the need for educational initiatives and resources for physicians to learn how to use social media platforms safely and effectively. Often, physicians discussed the need for “a teaching module: learn this, learn how to use this, do it this way…. I would be open to education” (participant 10). No differences in themes were identified between academic and community physicians.

### Facilitators to Social Media for Professional Purposes

When physicians were asked to describe facilitators to professional social media use, 3 main themes emerged, as follows: (1) networking, (2) education, and (3) promotion. The most commonly cited facilitator was the use of social media for networking and expanding professional interactions with colleagues. Several physicians reported varied uses for social media, describing social media to “be a very helpful vehicle for talking with other people in their area and disseminating… research findings” (participant 2). Another common theme was using social media to stay up to date with recent scientific findings. One physician “found [social media] to be a very good way to get information… about new thoughts or ideas or discussion from colleagues” (participant 2). The final key theme was the appeal of social media as a platform for physicians to publicize their academic and/or clinical work. Among physicians who used social media for promotion of their work, 1 physician described using social media “to highlight my own research… to bolster interest and enthusiasm” (participant 4).

### Barriers to Social Media Use for Professional Purposes

When physicians were asked to describe the barriers to social media use for professional purposes, 4 main themes emerged, as follows: (1) lack of experience and time, (2) uncertainty, (3) preference, and (4) blurred boundaries. The most commonly cited barrier to professional social media use was lack of experience and time. Physicians expressed reluctance to dedicate time to use and maintain social media: “I don’t have the time” (participant 36). The second most prominent theme was uncertainty of benefit. Physicians were hesitant to adopt social media use for professional purposes owing to the lack of evidence of benefit: “From a professional standpoint, I haven’t seen evidence of benefit” (participant 19). Third, physicians expressed preferences for alternative forms of professional communication: “I would rather have [patients] hear [my advice] from me directly [in person or via telephone] than hearing it on social media” (participant 32). The fourth most commonly cited barrier to professional social media use was physicians’ concerns about blurred boundaries and security and privacy issues: “I don’t want patients directly contacting me through social media…. There needs to be some fine line, and that gets crossed” (participant 1). Again, no differences in themes around facilitators and barriers were identified between academic and community physicians.

## Discussion

Increasing clinical trial accrual is a persistent challenge in oncology. Social media presents a potential opportunity to increase clinical trial awareness, promote patient understanding, and improve recruitment efforts. However, numerous barriers exist to the direct application of social media in therapeutic trial recruitment of patients with cancer. To our knowledge, this is the first study to provide physicians’ insights on this important topic.

There are several important implications of this study. First, our findings revealed that physicians are not currently comfortable with or prepared to effectively use social media for cancer clinical trial recruitment. Although there are some aspects of these new modes of communication that physicians are enthusiastic about (ie, increased visibility and awareness), several important concerns remain. Notably, many physicians felt uncomfortable with the idea of using social media because of increased administrative burden and concerns that the complexities of clinical trials would not be appropriately communicated. Additionally, physicians raised concerns about the difficulty of regulating social media and maintaining online privacy and ethical conduct.

Second, our work contributes to the current literature, which has acknowledged that using social media for recruitment has potential limitations related to issues of privacy, confidentiality, transparency of health information, the quality and reliability of posted information, and potential for coercion.^34,35^ As suggested by our results, the solution may be dependent on resources from institutional and/or national organizations to guide and support recruitment efforts using social media. Such efforts are already underway, evidenced by work by the American Society of Clinical Oncology to develop a social media guidance document, a formal policy, and an online course as well as to assemble a working committee to encourage digital engagement in the cancer community.^11,17^ Additional resources, such as institutional guidelines, tutorials, and social media strategists (ie, personnel who develop and manage targeted social media campaigns), may also help encourage investigators to engage in digital communication.

Third, additional research is needed to further examine and better understand the risks and benefits of using social media for clinical trial recruitment, better characterize physicians’ concerns among larger cohorts, and identify methods to optimize social media use in clinical trials. Importantly, the use of social media for clinical recruitment has potential implications in promoting the engagement of otherwise underrepresented groups. Research has shown that the gap between those who have access to digital technology and those who don’t has become increasingly narrow over time.^36^ This is important because increasing access to and understanding of research can play a part in reducing health disparities among ethnic minorities and disadvantaged populations.^37^

Finally, our findings show that physicians are interested in exploring ways to integrate social media in professional settings but reported several barriers to using social media for professional purposes. New technologies have transformed medicine and how health information is disseminated, and it is important to understand how physicians view and use these technologies for clinical care in the digital age. While there is perceived value in social media for networking and educational purposes, lack of time and blurred boundaries remain key barriers. Further research is needed to better understand physicians’ preferences regarding and motivations behind their use of social media for professional purposes.

### Limitations

There are several limitations to this study. First, there is limited generalizability of our findings, given that this study was conducted with physicians at COH, a comprehensive cancer center. City of Hope has a dedicated interest in conducting research; thus, the views expressed in this study may not be representative of all physicians in the United States or across diverse practice settings. However, we attempted to capture a broad range of perspectives by including physicians from 6 COH-affiliated community sites across Southern California. Second, our findings may not be representative of community sites that are not supported by an academic center. The community physicians who participated in this study are affiliated with the COH main campus, which provides support to community sites to conduct clinical trials. Third, nonphysician research personnel (ie, research nurses and research coordinators) were not included in this study. Future studies should seek insight from such individuals, who are often largely involved in the recruitment process. Fourth, member checking was not performed. Fifth, this study is a qualitative analysis, and therefore, findings are exploratory and hypothesis generating. We are unable to make claims beyond reporting the perceptions of participants. Despite this limitation, this study provides important insights that can help inform clinical researchers seeking to integrate social media in clinical trials and address persistent challenges to recruitment. It highlights the advantages of social media use and identifies key barriers that may be addressed to promote the use of social media for clinical trial recruitment. Further research is needed to address potential concerns that may arise in the future and gain a more comprehensive understanding of the risks and benefits that social media poses in clinical settings. Before social media can be integrated into clinical trials, specific guidelines must be defined for such use. Additionally, physicians must be provided tools with which to safely and effectively use social media in this way.

## Conclusions

Our findings reflect an open dialogue among physicians about using social media to enhance cancer clinical trial recruitment. While social media is a promising method of communicating clinical trial information to large and diverse populations, the idea of using social media for recruitment purposes poses challenges owing to physician concerns about increased administrative burden and the risk of misinformation. Further research is needed to examine how tailored interventions (eg, training, institutional resources and support, or policy) can address the concerns that physicians have with using social media to facilitate recruitment to cancer clinical trials. The information from this study can help guide the development of novel, targeted approaches to better inform investigators about how to safely and effectively use social media to increase clinical trial recruitment in oncology. Importantly, findings from this study may provide a road map for further research to use social media to improve clinical trial recruitment, not only for oncology, but across other specialties.

## Notes

Supplement. **eAppendix.** Semistructured Interview Guide
