# Supplementary material for: Physician Perceptions of the Use of Social Media for Recruitment of Patients in Cancer Clinical Trials
Source: JAMA Netw Open. 2019 Sep 18;2(9):e1911528. doi: 10.1001/jamanetworkopen.2019.11528 (PMC6751756; doi:10.1001/jamanetworkopen.2019.11528)
Supplement: Supplement. — eAppendix. Semistructured Interview Guide [file jamanetwopen-2-e1911528-s001.pdf]

Supplementary Online Content

Sedrak MS, Sun V, Liu J, et al. Physician perceptions of the use of social media for recruitment of patients in cancer clinical trials. *JAMA Netw Open*. 2019;2(9):e1911528. doi:10.1001/jamanetworkopen.2019.11528

eAppendix. Semistructured Interview Guide

This supplementary material has been provided by the authors to give readers additional information about their work.

© 2019 Sedrak MS et al. *JAMA Network Open.*

eAppendix. Semistructured Interview Guide

Introduction

Thank you for agreeing to participate in this interview study.

We are interested in learning more about your experiences as a medical oncologist who is involved in clinical trials. Your opinions are extremely valuable to us as we look for ways to improve clinical trial participation.

I will be recording the interview. Your responses will be recorded digitally and transcribed by a professional service, removing personal information before analysis to ensure confidentiality. All digital audio files will be deleted after transcription. I would ask you to please refrain from using any patient-identifying information during our conversation.

Your participation is entirely voluntary and you may refuse to participate or discontinue the interview at any time without penalty. By completing the interview, you have consented to our use of the information you provide.

Do you have any questions before we begin?

If you agree, I will now turn on the recorder and we will begin the interview.

[Interviewer will turn on the recorder.]

The recorder is now on.

First, I'd like to know about your general experience with cancer clinical trials.

1. How many years have you been practicing as a medical oncologist?

2. Approximately how many patients are in your patient panel? How many of your patients have ever participated or currently participate in a clinical trial?

I'd like to ask some questions about your perceptions of social media in relation to this problem. For our discussion, social media is defined as websites and application that enable users to create and share content, such as Facebook, Twitter, YouTube, etc.

3. Are you aware of social media being used to recruit patients to clinical trials? If yes, can you expand on your answer?

4. On a scale of 1 (not at all) to 5 (very much), how would you rate your interest in using social media for clinical trial recruitment? What about your comfort? How would you rate your concern about its use in this way? Would you like to expand on your answers?

© 2019 Sedrak MS et al. *JAMA Network Open.*

5. Do you use social media for personal purposes? Why or why not?

6. Do you use social media for professional purposes (e.g., networking, discussing relevant research in the field)? Why or why not?

7. Is there anything else that you would like to share with us on clinical trial enrollment, and use of social media in clinical trial?

Thank you again for your contribution. If you have any questions, please feel free to contact us using the contact information provided on the Statement of Research.

© 2019 Sedrak MS et al. *JAMA Network Open.*
